# Supplementary material for: Perspectives on healthy eating practices and acceptance of WIC-approved foods among parents of young children enrolled in WIC
Source: PLoS One. 2023 Dec 21;18(12):e0295902. doi: 10.1371/journal.pone.0295902 (PMC10734995; doi:10.1371/journal.pone.0295902)
Supplement: Attachment — Submitted filename: PONE-D-22-35532.pdf [file pone.0295902.s002.pdf]

PLOS ONE

Perspectives on healthy eating practices and acceptance of WIC-approved foods among parents of young children enrolled in WIC

--Manuscript Draft--

**Manuscript Number:**	PONE-D-22-35532
**Article Type:**	Research Article
**Full Title:**	Perspectives on healthy eating practices and acceptance of WIC-approved foods among parents of young children enrolled in WIC
**Short Title:**	A qualitative analysis of formative research
**Corresponding Author:**	Nour Hammad<br>Harvard University<br>Boston, MA UNITED STATES
**Keywords:**	Special Supplemental Nutrition Program for Women, Infants, and Children; Childhood Obesity; Maternal Nutrition; Low-Income; Food Preferences; Text Messaging; Nutrition Intervention
**Abstract:**	The prevalence of childhood obesity remains high in the US, particularly among children living in low-income households. Diet quality plays an important role in obesity prevention, particularly among mothers as they serve as role models. Those served by the Special Supplemental Nutrition Program for Women, Infants, and Children (WIC) receive nutrient rich foods aimed at increasing diet quality, yet redemption is low. Digital interventions targeting WIC parents show potential for behavior change and could be used for childhood obesity prevention. This study aims to describe the formative research conducted to understand perspectives on healthy eating practices, acceptance of WIC-approved foods, and preferences for the use of digital tools to improve the purchasing and consumption of WIC-approved foods to improve diet quality. In-depth interviews were conducted with 13 WIC parents and caregivers. A text messaging program, that includes sending weekly messages, recipes, and nutrition tips should be pilot tested as it is hypothesized to improve diet quality and increase redemption of WIC-approved foods.
**Order of Authors:**	Nour Hammad
	Melissa Kay
**Additional Information:**	
**Question**	**Response**
**Financial Disclosure**<br><br>Enter a financial disclosure statement that describes the sources of funding for the work included in this submission. Review the submission guidelines for detailed requirements. View published research articles from *PLOS ONE* for specific examples.<br><br>This statement is required for submission and **will appear in the published article** if the submission is accepted. Please make sure it is accurate.	M.K. received the Duke Center for Research to Advance Healthcare Equity (REACH Equity) award, which is supported by the National Institute on Minority Health and Health Disparities under award number U54MD012530. The funders had no role in study design, data collection and analysis, decision to publish, or preparation of the manuscript.

Powered by Editorial Manager® and ProduXion Manager® from Aries Systems Corporation

**Unfunded studies**<br>Enter: *The author(s) received no specific funding for this work.*<br><br>**Funded studies**<br>Enter a statement with the following details:<br>• Initials of the authors who received each award<br>• Grant numbers awarded to each author<br>• The full name of each funder<br>• URL of each funder website<br>• Did the sponsors or funders play any role in the study design, data collection and analysis, decision to publish, or preparation of the manuscript?<br>• **NO** - Include this sentence at the end of your statement: *The funders had no role in study design, data collection and analysis, decision to publish, or preparation of the manuscript.*<br>• **YES** - Specify the role(s) played.<br><br>* typeset	
**Competing Interests**<br><br>Use the instructions below to enter a competing interest statement for this submission. On behalf of all authors, disclose any competing interests that could be perceived to bias this work—acknowledging all financial support and any other relevant financial or non-financial competing interests.<br><br>This statement is required for submission and **will appear in the published article** if the submission is accepted. Please make sure it is accurate and that any funding sources listed in your Funding Information later in the submission form are also declared in your Financial Disclosure statement.<br><br>View published research articles from *PLOS ONE* for specific examples.	The authors have declared that no competing interests exist.

**NO authors have competing interests**<br><br>Enter: *The authors have declared that no competing interests exist.*<br><br>**Authors with competing interests**<br><br>Enter competing interest details beginning with this statement:<br><br>*I have read the journal's policy and the authors of this manuscript have the following competing interests: [insert competing interests here]*<br><br>* typeset	
**Ethics Statement**<br><br>Enter an ethics statement for this submission. This statement is required if the study involved:<br><br>• Human participants<br>• Human specimens or tissue<br>• Vertebrate animals or cephalopods<br>• Vertebrate embryos or tissues<br>• Field research<br><br>Write "N/A" if the submission does not require an ethics statement.<br><br>General guidance is provided below. Consult the submission guidelines for detailed instructions. **Make sure that all information entered here is included in the Methods section of the manuscript.**	All study procedures were approved by the ethical review board at Duke University and written informed consent was obtained from all participants.

**Format for specific study types**<br><br>**Human Subject Research (involving human participants and/or tissue)**<br>• Give the name of the institutional review board or ethics committee that approved the study<br>• Include the approval number and/or a statement indicating approval of this research<br>• Indicate the form of consent obtained (written/oral) or the reason that consent was not obtained (e.g. the data were analyzed anonymously)<br><br>**Animal Research (involving vertebrate animals, embryos or tissues)**<br>• Provide the name of the Institutional Animal Care and Use Committee (IACUC) or other relevant ethics board that reviewed the study protocol, and indicate whether they approved this research or granted a formal waiver of ethical approval<br>• Include an approval number if one was obtained<br>• If the study involved *non-human primates*, add *additional details* about animal welfare and steps taken to ameliorate suffering<br>• If anesthesia, euthanasia, or any kind of animal sacrifice is part of the study, include briefly which substances and/or methods were applied<br><br>**Field Research**<br><br>Include the following details if this study involves the collection of plant, animal, or other materials from a natural setting:<br>• Field permit number<br>• Name of the institution or relevant body that granted permission	
**Data Availability**<br><br>Authors are required to make all data underlying the findings described fully available, without restriction, and from the time of publication. PLOS allows rare exceptions to address legal and ethical concerns. See the PLOS Data Policy and FAQ for detailed information.	No - some restrictions will apply

A Data Availability Statement describing where the data can be found is required at submission. Your answers to this question constitute the Data Availability Statement and **will be published in the article**, if accepted.<br><br>**Important:** Stating 'data available on request from the author' is not sufficient. If your data are only available upon request, select 'No' for the first question and explain your exceptional situation in the text box.<br><br>Do the authors confirm that all data underlying the findings described in their manuscript are fully available without restriction?	
**Describe where the data may be found in full sentences. If you are copying our sample text, replace any instances of XXX with the appropriate details.**<br><br>• If the data are **held or will be held in a public repository**, include URLs, accession numbers or DOIs. If this information will only be available after acceptance, indicate this by ticking the box below. For example: *All XXX files are available from the XXX database (accession number(s) XXX, XXX.)*.<br>• If the data are all contained **within the manuscript and/or Supporting Information files**, enter the following: *All relevant data are within the manuscript and its Supporting Information files.*<br>• If neither of these applies but you are able to provide **details of access elsewhere**, with or without limitations, please do so. For example:<br><br>*Data cannot be shared publicly because of [XXX]. Data are available from the XXX Institutional Data Access / Ethics Committee (contact via XXX) for researchers who meet the criteria for access to confidential data.*<br><br>*The data underlying the results presented in the study are available from (include the name of the third party*	Data cannot be shared publicly because of their sensitivity (e.g., contact information of participants in the WIC assistance program). Only the research team has access to the data, which are stored on a secure password-protected Box folder.

and contact information or URL).<br>• This text is appropriate if the data are owned by a third party and authors do not have permission to share the data.<br><br>* typeset	
Additional data availability information:	

Manuscript
Click here to access/download;Manuscript;Manuscript - Qualitative Anlaysis.docx

Perspectives on healthy eating practices and acceptance of WIC-approved foods among parents of young children enrolled in WIC

Nour M Hammad[1,*] and Melissa C Kay[2]
[1]Harvard T.H. Chan School of Public Health, 677 Huntington Ave, Boston, MA 02115
[2]Duke University, 3116 N. Duke Street, Durham, NC 27704

*Corresponding author
E-mail: nourhammad@fas.harvard.edu

Acknowledgements: We thank Jennifer Headley and Dr. Mary Story at Duke University for their valuable input.

Funding: This study was supported by the Duke Center for Research to Advance Healthcare Equity (REACH Equity), which is supported by the National Institute on Minority Health and Health Disparities under award number U54MD012530. The funders had no role in study design, data collection and analysis, decision to publish, or preparation of the manuscript.

Conflict of Interest: The authors declare that they have no conflicts of interest.

Contributions: NMH and MCK - conceptualization and formal analysis. NMH - Writing – Original Draft Preparation. MCK - Writing – Review & Editing and Supervision. NMH and MCK have read and approved the manuscript.

Ethical Statement: All study procedures were approved by the ethical review board at Duke University and written informed consent was obtained from all participants.

Abstract

The prevalence of childhood obesity remains high in the U[illegible]articularly among children living in low-income households. Diet quality plays an important role in obesity prevention, particularly among mothers as they serve as role models. Those served by the Special Supplemental Nutrition Program for Women, Infants, and Children (WIC) receive nutrient rich foods aimed at increasing diet quality, yet redemption is low. Digital interventions targeting WIC parents show potential for behavior change and could be used for childhood obesity prevention. This study aims to describe the formative research conducted to understand perspectives on healthy eating practices, acceptance of WIC-approved foods, and preferences for the use of digital tools to improve the purchasing and consumption of WIC-approved foods to improve diet quality. In-depth interviews were conducted with 13 WIC parents and caregivers. A text messaging program, that includes sending weekly messages, recipes, and nutrition tips should be pilot tested as it is hypothesized to improve diet quality and increase redemption of WIC-approved foods.

Keywords:

Special Supplemental Nutrition Program for Women, Infants, and Children, Childhood Obesity, Maternal Nutrition, Low-Income, Food Preferences, Text Messaging, Nutrition Intervention

Introduction

Nearly 20% of children in the U.S. have obesity, affecting 14.4 million children [1]. Childhood obesity puts children at a higher risk of developing metabolic syndrome, type 2 diabetes, kidney diseases, and cardiovascular diseases later in adulthood [2, 3]. Childhood obesity is also associated with stigma, depression, anxiety, lower self-esteem, school absenteeism and poorer academic performance [4-6]. These long- and short-term implications underscore the importance of preventing childhood obesity. Studies show that family-based interventions, especially those targeting mothers, can be facilitators of decreasing childhood obesity from the early stages of child development [7-9]. Mothers heavily influence their child's intake, as they are often gatekeepers of food in the home. They play a critical role in their children's eating behavior through role modeling, creating food associations, and providing access to certain types of food [10, 11].

The Special Supplemental Nutrition Program for Women, Infants, and Children (WIC) serves pregnant, postpartum, and breastfeeding women, infants, and children up to age 5 years who live in low-income households. WIC provides vouchers for healthy foods; nutrition education; breastfeeding support; and medical and social-service referrals [12]. A hallmark of the WIC program is the food packages which are designed to meet the specific nutritional needs of WIC beneficiaries and align with national dietary guidance. The food packages include vouchers for nutrient rich foods like fruits and vegetables, low-fat/skim milk, and whole-grain products. Studies show that participation in WIC positively influences diet quality and rates of overweight and obesity [13, 14]. However,

WIC has experienced a decline in enrollment, retention, and food package redemption over the past decade [15, 16]. In addition to structural barriers associated with enrollment, dissatisfaction with the retail experience and confusion over which products are included contribute to decreased redemption of WIC-approved foods [17, 18]. However, little is known about how WIC participants view healthy eating and how WIC-approved foods are, or are not, embedded into the family diet. Given the impacts WIC has on childhood obesity and diet quality, efforts are needed to support the reach and satisfaction of WIC participants to maximize program efficacy.

Smartphone ownership is ubiquitous in the U.S. and particularly high among those living in low-income households [19-21]. Digital interventions that focus on parents' behaviors, specifically mothers, have proven to be efficacious and have a positive impact on childhood obesity [22-24]. Yet few have aimed to improve diet quality, particularly among low-income populations [25]. Thus, this study describes the formative work conducted to develop a digital behavioral intervention for parents and caregivers enrolled in WIC to improve maternal diet quality by supporting redemption of WIC-approved foods [26]. To ensure the intervention is congruent with the needs of WIC parents and caregivers, we conducted in-depth interviews to guide the development. These interviews focused on highlighting barriers and facilitators to consuming WIC-approved foods and behaviors associated with healthy eating. The aim of this paper is to describe findings from the formative research and provide guidance for future intervention development.

Materials and methods

The 21-item Standards for Reporting Qualitative Research (SRQR) checklist (S1 Appendix) was used to ensure transparency in conducting this research and reporting its results.

a. Recruitment

Recruitment focused on WIC participants receiving benefits from a network of federally qualified health centers that serves medically vulnerable patients throughout central North Carolina. Specific details on the recruitment procedure are reported elsewhere [26]. Briefly, WIC parents and caregivers were recruited by WIC clinicians during their regularly scheduled appointments. WIC clinicians recorded contact information for interested WIC parents (i.e., participants) using an online study form securely stored in a password-protected Box folder that could only be accessed by the research team. The research team followed up with those who were interested and assessed eligibility. If eligible, the parent completed an online consent form and baseline survey and scheduled a date and time for the interview.

b. Participants

Recruitment occurred from May 2020 through August 2020. Eligibility criteria included the following: parent or caregiver with a child 2 years old or younger, receiving benefits from WIC, have a cell phone that can receive text messages, and are English speakers. We enrolled 13 parents and caregivers of children receiving WIC benefits. A larger sample size was planned, but we faced significant recruitment challenges due to the

COVID-19 pandemic. Though the sample is small, prior work demonstrates it is sufficient for understanding common perceptions and experiences among a relatively homogenous group [27]. The flow of participants through the formative research is presented in Fig 1.

Fig 1. Consort diagram describing flow of participants through the formative research

c. Procedures

We conducted in-depth, semi-structured interviews [28] to obtain detailed information on a set of topics and explore food preferences and habits [29, 30]. An interview guide was developed by MCK, the principal investigator, with input from stakeholders including WIC clinicians and content experts in qualitative research. The interview guide consisted of open-ended questions related to redemption and use of foods received in WIC packages; thoughts about and practice of healthy eating; and WIC's role in promoting healthy eating. Other questions included thoughts about digital interventions (see Table 1).

Table 1. Topics included in the interview guid[illegible]

Participation in WIC	
	Foods liked
	Foods disliked
	Most purchased food items
	Least purchased food items
Healthy Eating	
	Definition of healthy eating

Cooking methods
WIC’s helpfulness in healthy eating promotion
Source of nutrition information
Text Messaging
Helpfulness of text messaging
Frequency of receiving text messages

Digitally audio-recorded interviews (n=13) were conducted between June and September 2020. The interviews were on average 25 minutes long (range 13 to 39 minutes). All interviews were conducted by trained researchers. Participants were compensated with a $25 Walmart gift card. All interviews were transcribed by a professional transcription agency and verified for accuracy. All study procedures were approved by the ethical review board at Duke University.

d. Analysis

We used an in-depth reflexive thematic approach to data analysis to identify, analyze, and report themes within data [31]. Mostly structural codes were used to create a codebook containing 14 sub-themes clustered into four themes. NH and MCK each independently coded the interviews using NVivo12 [32]. Although inter-coder reliability is discouraged with the reflexive thematic approach, we conducted it to assess the need for improvement in the quality and credibility of the coding process [33, 34]. Any individual code that had a kappa below 0.81 [35] was reviewed as a team and every discrepancy was discussed and corrected. This was feasible given the small sample size. Agreement was met on every code and the final overall kappa was 0.96.

Results

a. Description of the sample

Most of the participants were the child's mother (n=12); 1 was the grandmother (Table 2). Participants had a mean age of 31 years (range 23 - 45), approximately two thirds (n=8) identified as white, with one identifying as white and Hispanic/Latina; the remaining five participants identify as non-Hispanic Black. About half (n=7) had some college education or obtained a college degree, with five participants graduating from high school.

Table 2. Characteristics of the participants who completed interviews (n=13).

**Age, mean in years (range)**		31.4 (23.0-45.0)
**Race/Ethnicity, % (n)**	**Black**	38.5 (5)
	**White only**	53.8 (7)
	**White and Hispanic/Latina**	7.7 (1)
**Education, % (n)**	**Some high school**	7.7 (1)
	**High school**	38.5 (5)
	**Some college**	30.8 (4)
	**≥ College degree**	23.0 (3)
**Married, % (n)**		30.8 (4)
**Employed*, % (n)**		38.5 (5)

*Both part-time (n=2) and full-time employment (n=3)

b. Qualitative findings

The qualitative findings are presented below. Theme 1 focuses on healthy eating; theme 2 focuses on the purchase of WIC-approved foods; theme 3 focuses on WIC's role in promoting healthy eating; and theme 4 discusses participants' thoughts about a text messaging program designed to improve healthy eating, its content, and frequency of receiving text messages. Salient quotes were used to illustrate each sub-theme.

Theme 1: (Un)healthy Eating

Participants defined their perspectives of healthy and unhealthy eating, their sources of nutrition information, and how they practice healthy eating.

Healthy eating definitions and practices

Healthy eating has different definitions according to different individuals. About half (n=6) reported that daily consumption of fruits and vegetables encompasses healthy eating. Other definitions included portion control; moderation; balance and variety of foods when eating; eating fresh, unprocessed, organic and/or whole foods; eating proteins; whole grain foods; eating foods that help with digestion; and staying hydrated.

> *If you know how to control it and you don't intake too much junk food. It is not hard to have a healthy diet and enjoy some junk foods every once in a while.* (29-year-old, white mother)

I think healthy is the less that you have to do to it [unprocessed] … the healthier it is. (31-year-old white mother)

About half (n=6) reported trying to include fruits and vegetables with all their – and their children's – meals on a daily basis. Other non-frequently reported practices of healthy eating included finding healthier ways to prepare meals such as grilling and baking instead of frying, using cast iron pans, eating in moderation, following certain eating patterns such as intermittent fasting, eating organic foods, comparing food labels, eating fresh foods instead of canned or frozen foods, and sticking to routines.

Go to the event and if there's food there, you want to eat, eat it, but eat small portions of it. (45-year-old, white grandmother)

Unhealthy eating definitions and practices

Participants were asked to specify definitions of foods or behaviors that are considered unhealthy. Most participants (n=10) mentioned high sugar foods or foods containing added sugars as unhealthy, such as sodas, juice, and candies. As a 34-year-old Black mother said, "Things that I like ... Like cake, ice cream, chocolate." According to participants, this definition of unhealthy eating contributes to negative health effects on the body like the effect of sugary beverages on one's dental health.

Many participants (n=7) considered high sodium foods and processed foods to be unhealthy, such as canned foods, chips, macaroni and cheese, and hotdogs. For example,

a 30-year-old, white and Hispanic/Latina mother, said “Canned food is very high in sodium. Sodium is not good for your body.”

Other examples of unhealthy food definitions included fast foods (n=4), and overconsumption of any foods (n=2). One 45-year-old, white grandmother reported whole wheat foods as unhealthy, believing it raises blood glucose and insulin levels. Another participant reported foods that are labeled as “light” as unhealthy because of their aspartame content.

About half (n=6) reported avoiding high salt and processed foods, frozen meals, and high sugar foods and beverages. Other practices included avoiding junk food, reading food labels, and diluting juices.

> *I thought that I was getting something healthy because it was a little snack bar for my son and then I looked at the sugar and it was like 25% of your daily value and it was just such a small little snack bar. And I was like, "Oh my gosh, this is really bad.”* (27-year-old, white mother)

Source of nutrition information

Participants highlighted many different sources for getting information about nutrition, healthy eating, and meal preparation. Many participants (n=7) reported obtaining their nutrition information from online resources such as Google, YouTube, Pinterest, and Facebook. Some (n=5) also referred to WIC nutritionists who would talk about healthy eating during their appointments or refer them to resources that help with

meal preparation. Other cited recipes, friends and family, food labels, partners, cooking shows, and research. One participant also mentioned that she is her own source of information because she relies on her common sense.

Devices used to get information about healthy eating

When asked if they use their phone, computer, or tablet to get information about healthy eating, many (n=7) mentioned using their phones for convenience and ease. Some (n=4) mentioned using their computers, along with their phones, except for one participant who reported using her computer exclusively. Only one participant mentioned using a tablet, along with her phone and computer.

I just use my phone for everything… Yeah, it's just easier, it's right in your hand. (29-year-old, white mother)

Theme 2: Purchase of WIC-approved foods

Participants reported the least and most purchased WIC-approved foods, and the barriers and facilitators, respectively, affecting these purchasing behaviors.

Most purchased foods and facilitators to these purchases

Fruits, vegetables, milk, cheese, and eggs were the most purchased WIC foods as presented in the order in table 3.

Table 3. Most purchased WIC-approved foods (n=13).

Foods most purchased	Number of participants who reported these foods
Fruits	12
Vegetables	11
Milk	11
Cheese	11
Eggs	8
Bread	8
Cereal	7
Peanut butter	6
Juice	5
Yogurt, Fish, Beans	<5

The most cited reason for purchasing these foods was that the foods are used or liked by the children and/or themselves. Other reasons included that the foods are healthy, tasty, and free, and that there are many options that are provided such as lactose free milk and soy milk. As a 30-year-old, white and Hispanic/Latina mother said, “Because it's healthier eating for me and my kids.”

Least purchased foods and barriers to these purchases

Yogurt and peanut butter were reported as the least purchased foods as presented in table 4.

Table 4. Least purchased WIC-approved foods (n=12).

Foods least purchased	Number of participants who reported these foods
Yogurt	6
Peanut butter	3

Bread	3
Cereal, Beans, Fish, Milk, Juice, Eggs, Brown Rice, Canned Foods, Fruits, Vegetables	<3

The biggest barrier for purchasing WIC-approved foods was that their children are picky eaters and dislike the foods (n=8). Only 2 participants reported not purchasing WIC-approved foods because they dislike those foods. Other barriers included the low amount of money allocated for purchasing fruits and vegetables; small in-store labeling; big food sizes leading to food waste; children's health conditions; limited options of WIC-approved foods; the negative health effects of some of WIC-approved foods (e.g., juice); and the unfamiliarity to cook certain foods.

> *Sometimes I have trouble figuring out what brands of tortillas and other whole wheat stuff places are participating in WIC with because only Food Lion will put up the WIC signs and I haven't seen WIC signs in other stores. Like, Walmart is closer to us and they don't have their signs*. (27-year-old, white mother)

Theme 3: WIC's role in healthy eating

When asked about the role WIC plays in promoting healthy eating, participants presented a variety of ideas as well as how WIC helps with other matters.

WIC's helpfulness in promoting healthy eating

All participants believed that WIC helps them eat healthier because WIC provides only healthy foods in their food packages.

> *It gives access to some healthy items that we need daily*. (29-year-old, white mother)

Another reason reported was that WIC nutritionists are nice and helpful; they are a great source for credible nutrition information about healthy eating and they sometimes help refer participants to other resources that can provide them with recipes, snack ideas, and physical activity recommendations.

> *I've never met anybody that was not that nice and did not sit there and listen to me and hear me out.* (29-year-old, white mother)

The third most cited reason was WIC's resources, including educational pamphlets, shopping guide, MyPlate, and food models, which help with portion control. Other reasons included that WIC allows participants to try new food items such as soy milk and lactose-free milk that they wouldn't buy otherwise.

> *Yes [it's helpful], because it [WIC] gives you a lot of information and a lot of resources if you have questions.* (29-year-old, Black mother)

Two participants shared that although they consider WIC to be helpful in promoting healthy eating, they think WIC can do better. That is because although WIC offers healthy foods, they do not offer guides to help them cook or use these foods. As one 34-year-old, Black mother shared, nothing can help one eat healthy unless they are willing to: “It's just a mind over matter thing for me as far as how I'm going to eat healthy because I can eat healthy without WIC or with it…” Another participant believed that WIC does not offer healthy foods as she believes that whole wheat foods are not healthy.

Other benefits of WIC

Few participants (n=3) mentioned that WIC is helpful in ways others than promoting healthy eating. Some noted that WIC helps by providing formula which is expensive, breastfeeding classes, and ways to address their children’s picky eating or feeding transitions.

> *That [Formula] is definitely a blessing to me because a can of milk is expensive… But also the lady [WIC nutritionist] that I go to, she gives me like different eating habits and when to know how to transition from the bottle to the sippy cup. So, those are helpful*. (34-year-old, Black mother)

WIC’s material vs general online material

Six participants commented on the comparison between the material WIC offers (e.g., its website, physical material such as brochures, and appointments with WIC

nutritionists) and online material (e.g., Google). Two of these participants could not make a comparison, because they do not use WIC's material.

> *I haven't really explored it [WIC's website] to even understand it may have recipes on there or it may have other things that I could use for dinner.* (34-year-old, Black mother)

Two believed that WIC is more helpful because you can get answers during your appointment with WIC nutritionists and because WIC focuses more on healthy food items compared to online material.

> *I guess some of the stuff that a nutritionist does, I could also just look up online on different blogs for toddlers and babies. So it's not really that different but it's a little more helpful to have a conversation with someone.* (27-year-old, white mother)

Two believe that online information is better, because although WIC provides healthy foods, online sources provide information on how to utilize them.

They give me just the staple foods, whereas online would give me a little bit more information on how to make things, portion sizes, stuff like that. (23-year-old, white mother)

Ways WIC can help

Five participants said they wish WIC could provide them with more money for fruits and vegetables to help them last for the entire month. Of note, during the pandemic, but after these interviews were collected, WIC increased the fruits and vegetables benefit from $7-11 to $24-47 per month [36].

With my WIC package, I only get $9 for fruits and vegetables. And I mean, in this day and age, that's not really a lot to do with what fruits and vegetables you can get. (27-year-old, white mother)

About half (n=5) also said that WIC can help by providing more food options such as almond milk or vegetable smoothies and healthier cereals. Other wishes were that WIC could send recipes to parents, update their pamphlets, offer organic options, focus more on parents, offer smaller sizes of foods, offer proteins such as chicken, and instruct stores to have bigger labels.

WIC could offer some type of weekly or monthly healthy recipe little sheet or something. Maybe once a month, they could pick up a recipe and send it out through emails or something. (29-year-old, white mother)

I think it's a waste, because we don't eat a whole lot of yogurt, so it just sits in my refrigerator and then goes bad I throw it out … They have a one size fits all deal, and that just doesn't work when you're talking about food and different people's body types and what their health condition and what's going on in their bodies. (45-year-old, white grandmother)

Theme 4: Text Messaging Program to improve healthy eating

When discussing a text messaging program aimed at improving healthy eating, participants generally had positive feedback.

Thoughts about the Program

Most (n=11) believed that developing a text messaging program aimed to improve intake is a good idea, as long as it provides non-repetitive information and gives participants the option to opt out.

Maybe they work from home and they use their phone for work. And if they're already getting a lot of emails and texts about work or something else, they may

feel like it's overwhelming, but I think it would be a good idea. (39-year-old, Black mother)

Two participants compared receiving text messages to emails and believe that receiving texts is easier than emails.

A lot of times people don't check their emails so I feel the texts would be more accessible. (27-year-old, Black mother)

Content of the program

Most (n=8) wanted recipes and some specifically mentioned healthy, inexpensive, and quick recipes or recipes for foods in season. The second most common content reported was tips and nutrition suggestions for both kids and parents (n=6). Other ideas included giving examples of health benefits of foods provided in WIC packages, healthy eating habits that parents can follow, portion control, moderation, balance in meal preparation, motivational messages, research, and anything that is considered informational.

Because sometimes with a limited selection of things, it's hard to come up with new ideas of what to make with the food. (27-year-old, white mother)

Frequency of receiving text messages

When asked about the frequency of the text messages, most (n=10) preferred to receive text messages once a week, as frequencies more than once might get overwhelming. Around half (n=5) suggested receiving text messages in the morning, which could serve as a reference if they go grocery shopping later or to get ideas for what to cook for dinner. They can also read the messages while they’re on their way to work.

> *Just because I'm up. I have to work. So, I am constantly doing things. I may not have time to even talk, but I can look at my phone and read an article ahead of time versus at night, taking care of her. It's more of a downtime.* (34-year-old, Black mother)

> *So I have time to think about during the day and then I'd be able to think about a dinner plan.* (23-year-old, white mother)

Discussion

Interviewing a sample of caregivers of young children enrolled in WIC provided perspectives on healthy eating practices, preferences of WIC-approved foods, and ways WIC can improve support for healthy eating that can be used to guide future programs. The results demonstrate a variety of definitions for healthy (and unhealthy) eating that exist among caregivers participating in WIC. These definitions also contribute to

individuals’ practices of healthy eating as supported by previous research [37, 38]. However, surprisingly, in our sample only about half of participants reported eating fruits and vegetables as part of healthy eating practices. Results also highlight a variety of sources used to find nutrition information. These results confirm the need to understand perspectives on healthy eating to help in creating and delivering messages to inform behavior change [38, 39].

Uncovering the most and least purchased WIC-approved foods helps determine what WIC foods should be focused on to increase redemption rates and maximize program efficacy while developing the intervention. The most purchased food items identified were fruits, vegetables, milk, cheese, and eggs. The least purchased food items were yogurt and peanut butter. Our results are in alignment with a study conducted in 2018 that showed foods redeemed the most were milk, fruits, vegetables, and eggs and the foods redeemed the least included, beans, peanut butter, and whole grains [40]. Although our study had a smaller sample size, we identified cheese as another often purchased food and yogurt as one of the least purchased foods as opposed to whole grains. Using these results, future studies can focus on those foods participants are least likely to redeem by providing recipes and creative uses for such foods in meal and snacks throughout the day.

The biggest facilitator for purchasing WIC-approved foods was the preference of children and caregivers. However, the biggest barrier was children’s picky eating behaviors. This underscores the importance of addressing this reoccurring child behavior. An expert

panel report by the Healthy Eating Research national program suggests following these approaches to help address picky eating: parental modeling of healthy consumption, availability of healthy foods in the house, having family meals, and promoting food acceptance through repeated exposures to foods [41]. WIC can help by providing anticipatory guidance and education about these barriers and how to overcome them via educational material, communication during appointments with WIC nutritionists, and raising awareness about these barriers with providers who refer people to WIC.

Another barrier to full redemption of WIC-approved foods identified in this study is caregivers' dislike of some foods. Research has shown that the lack of flexibility in food packages to match individual preferences often results in benefits left unspent [42]. Other barriers include small in-store food labels, receiving excess amounts of foods, and unfamiliarity with certain foods' preparation. These findings align with those identified in recent studies [42, 43]. Another barrier to be considered is in-store grocery shopping during the COVID-19 pandemic; online grocery shopping has increased tremendously as a way to decrease transmission and adhere to safety precautions [44, 45]. However, there is no equitable access to online grocery shopping for WIC participants and structural changes to the WIC program are needed to make it more accessible to participants and optimize their shopping experience [46, 47].

WIC can further promote healthy eating by allocating more money towards purchasing fruits and vegetables and providing a larger variety of foods. The American Rescue Plan

Act enacted in March 2021 includes investments in WIC by temporarily increasing the participants' benefits for the purchase of fruits and vegetables in order to increase access to them which aims to improve the health, food security, and financial stability of WIC participants during the COVID-19 pandemic [48]. Future research should assess the perceived value of this increase in access to fruits and vegetables and expansion of WIC-approved food products. Furthermore, as a COVID-19 emergency response, WIC enacted some program flexibilities and provided its participants with COVID-19 food package waivers, allowing them to purchase a wider variety of foods [49]. Future studies should assess how such flexibilities impact the purchasing and healthy eating behaviors of participants [50].

Most caregivers reported using their phone to get nutrition information. Thus, a text messaging program could be used to improve healthy eating and WIC food redemption as it is well accepted by individuals. Similarly, in another study, texting was the most referenced feature used by WIC participants [51]. Text messaging interventions been successful in improving early post- delivery contact and exclusive breastfeeding rates among WIC mothers who receive peer counselling [52]. Our results suggest future interventions should consider using text messages that include recipes and nutrition tips to support WIC participants in making healthy behavior changes and improving the redemption of WIC-approved foods.

Strengths and limitations

The strength of this study resides in the duality of coding which underscores the credibility of the results presented, leaving less room for error. This study also has limitations. First, two clinical research coordinators were responsible for conducting most of the interviews, which can add some inconsistency in data collection. The small sample size of 13 participants may offer less generalizable results; most participants came from urban settings and immigrant groups were not represented as the study was conducted in English only due to staffing and budget restrictions.

Conclusion

The results of the formative research show the importance of communicating with the target audience before implementing an intervention to ensure that it is relevant and targeted to their needs. These findings can be coupled with previously published formative research to suggest an intervention aimed at improving WIC caregivers' healthy eating habits and improving redemption of WIC approved foods should include text messages with recipes and healthy eating tips. The findings also help inform ways to address barriers to redemption and improve caregivers' diet quality, which include tailoring educational material to these topics, raising awareness about these topics with WIC clinicians, and making structural changes to enable WIC participants to purchase WIC-approved foods online.

References

1. Fryar CD CM, Afful J. . Prevalence of overweight, obesity, and severe obesity among children and adolescents aged 2–19 years: United States, 1963–1965 through 2017–2018. 2020.

2. Nehus E, Mitsnefes M. Childhood obesity and the metabolic syndrome. Pediatric Clinics. 2019;66(1):31-43.

3. Singer K, Lumeng CN. The initiation of metabolic inflammation in childhood obesity. The Journal of clinical investigation. 2017;127(1):65-73.

4. Topçu S, Orhon FŞ, Tayfun M, Uçaktürk SA, Demirel F. Anxiety, depression and self-esteem levels in obese children: a case-control study. Journal of Pediatric Endocrinology and Metabolism. 2016;29(3):357-61. doi: doi:10.1515/jpem-2015-0254.

5. Taras H, Potts- Datema W. Obesity and student performance at school. Journal of School Health. 2005;75(8):291-5.

6. Puhl RM, Latner JD. Stigma, obesity, and the health of the nation's children. Psychological bulletin. 2007;133(4):557.

7. FiksAlexander G, GruverRachel S, Bishop-GilyardChanelle T, SuhAndrew W, KalraGurpreet K, DeRussoPatricia A, et al. A social media peer group for mothers to prevent obesity from infancy: the Grow2Gether randomized trial. Childhood Obesity. 2017.

8. French GM, Nicholson L, Skybo T, Klein EG, Schwirian PM, Murray-Johnson L, et al. An evaluation of mother-centered anticipatory guidance to reduce obesogenic infant feeding behaviors. Pediatrics. 2012;130(3):e507-e17.

9. Mustila T, Raitanen J, Keskinen P, Saari A, Luoto R. Lifestyle counselling targeting infant's mother during the child's first year and offspring weight development until 4 years of age: a follow-up study of a cluster RCT. BMJ Open. 2012;2(1):e000624. doi: 10.1136/bmjopen-2011-000624.

10. Scaglioni S, Arrizza C, Vecchi F, Tedeschi S. Determinants of children's eating behavior. The American journal of clinical nutrition. 2011;94(suppl_6):2006S-11S.

11. Mura Paroche M, Caton SJ, Vereijken CM, Weenen H, Houston-Price C. How infants and young children learn about food: A systematic review. Frontiers in Psychology. 2017;8:1046.

12. Kramer-LeBlanc CS, Mardis A, Gerrior S, Gaston N. Review of the nutritional status of WIC participants. 1999.

13. Daepp MI, Gortmaker SL, Wang YC, Long MW, Kenney EL. WIC food package changes: trends in childhood obesity prevalence. Pediatrics. 2019;143(5).

14. Chaparro MP, Crespi CM, Anderson CE, Wang MC, Whaley SE. The 2009 special supplemental nutrition program for women, infants, and children (WIC) food package change and children's growth trajectories and obesity in Los Angeles County. The American journal of clinical nutrition. 2019;109(5):1414-21.

15. Oliveira V. WIC participation continues to decline. Amber Waves. 2017.

16. Li X, McLaughlin PW, Saitone TL, Sexton RJ. The magnitude and determinants of partial redemptions of food benefits in the special supplemental nutrition program for women, infants and children (WIC). American Journal of Health Promotion. 2021;35(6):775-83.

17. Chauvenet C, De Marco M, Barnes C, Ammerman AS. WIC recipients in the retail environment: A qualitative study assessing customer experience and satisfaction. Journal of the Academy of Nutrition and Dietetics. 2019;119(3):416-24. e2.

18. Zimmer MC, Beaird J, Steeves EA. WIC Participants' Perspectives of Facilitators and Barriers to Shopping With eWIC Compared With Paper Vouchers. Journal of Nutrition Education and Behavior. 2021;53(3):195-203. doi: https://doi.org/10.1016/j.jneb.2020.09.007.

19. Alkhaldi G, Hamilton FL, Lau R, Webster R, Michie S, Murray E. The effectiveness of technology-based strategies to promote engagement with digital interventions: a systematic review protocol. JMIR research protocols. 2015;4(2):e47.

20. Statista. Percentage of U.S. adults who own a smartphone from 2011 to 2021 2021. Available from: https://www.statista.com/statistics/219865/percentage-of-us-adults-who-own-a-smartphone/.

21. Bensley RJ, Hovis A, Horton KD, Loyo JJ, Bensley KM, Phillips D, et al. Accessibility and preferred use of online Web applications among WIC participants with Internet access. Journal of Nutrition Education and Behavior. 2014;46(3):S87-S92.

22. Knowlden AP, Sharma M, Cottrell RR, Wilson BRA, Johnson ML. Impact Evaluation of Enabling Mothers to Prevent Pediatric Obesity Through Web-Based Education and Reciprocal Determinism (EMPOWER) Randomized Control Trial. Health Education & Behavior. 2015;42(2):171-84. doi: 10.1177/1090198114547816. PubMed PMID: 25161168.

23. Wen LM, Rissel C, Baur LA, Hayes AJ, Xu H, Whelan A, et al. A 3-arm randomised controlled trial of communicating healthy beginnings advice by telephone (CHAT) to mothers with infants to prevent childhood obesity. BMC Public Health. 2017;17(1):1-10.

24. Price S, Ferisin S, Sharifi M, Steinberg D, Bennett G, Wolin KY, et al. Development and implementation of an interactive text messaging campaign to support behavior change in a childhood obesity randomized controlled trial. Journal of health communication. 2015;20(7):843-50.

25. Ronteltap A, Bukman AJ, Nagelhout GE, Hermans RC, Hosper K, Haveman-Nies A, et al. Digital health interventions to improve eating behaviour of people with a lower socioeconomic position: a scoping review of behaviour change techniques. BMC nutrition. 2022;8(1):1-13.

26. Kay MC, Hammad NM, Herring SJ, Bennett GG. Using Interactive Text Messaging to Improve Diet Quality and Increase Redemption of Foods Approved by the Special Supplemental Nutrition Program for Women, Infants, and Children: Protocol for a Cohort Feasibility Study. JMIR research protocols. 2021;10(12):e32441.

27. Guest G, Bunce A, Johnson L. How many interviews are enough? An experiment with data saturation and variability. Field methods. 2006;18(1):59-82.

28. Guest G, Namey E, Mitchell M. In-depth interviews. Collecting Qualitative Data: A Field Manual for Applied Research [online]. 2013:113-71.

29. Johnstone M-L. Depth interviews and focus groups. Formative research in social marketing: Springer; 2017. p. 67-87.

30. Merritt R, Vogel M. Case Studies in Formative Research. Formative Research in Social Marketing: Springer; 2017. p. 281-90.

31. Braun V, Clarke V. Reflecting on reflexive thematic analysis. Qualitative research in sport, exercise and health. 2019;11(4):589-97.

32. QSR International Pty Ltd. (2020) NVivo (released in March 2020). Available from: https://www.qsrinternational.com/nvivo-qualitative-data-analysis-software/home.

33. Burla L, Knierim B, Barth J, Liewald K, Duetz M, Abel T. From Text to Codings: Intercoder Reliability Assessment in Qualitative Content Analysis. Nursing Research. 2008;57(2):113-7. doi: 10.1097/01.NNR.0000313482.33917.7d. PubMed PMID: 00006199-200803000-00007.

34. O'Connor C, Joffe H. Intercoder Reliability in Qualitative Research: Debates and Practical Guidelines. International Journal of Qualitative Methods. 2020;19:1609406919899220. doi: 10.1177/1609406919899220.

35. Altman D. Inter-rater agreement. Practical statistics for medical research. 1991:403-9.

36. : National WIC Association. 2021. Available from: https://www.nwica.org/blog/cvb-updated-amounts-toolkit#.YW23qxrMJPa?eType=EmailBlastContent&eId=44444444-4444-4444-4444-444444444444&eType=EmailBlastContent&eId=542ebcbc-2d52-4bd9-9aa6-891f0e5eae1c.

37. Lake AA, Hyland RM, Rugg-Gunn AJ, Wood CE, Mathers JC, Adamson AJ. Healthy eating: perceptions and practice (the ASH30 study). Appetite. 2007;48(2):176-82.

38. Falk LW, Sobal J, Bisogni CA, Connors M, Devine CM. Managing healthy eating: definitions, classifications, and strategies. Health education & behavior. 2001;28(4):425-39.

39. Ronteltap A, Sijtsema SJ, Dagevos H, de Winter MA. Construal levels of healthy eating. Exploring consumers' interpretation of health in the food context. Appetite. 2012;59(2):333-40.

40. Weber S, Uesugi K, Greene H, Bess S, Reese L, Odoms-Young A. Preferences and perceived value of WIC foods among WIC caregivers. Journal of nutrition education and behavior. 2018;50(7):695-704.

41. Evidence-Based Recommendations and Best Practices for Promoting Healthy Eating Behaviors in Children 2 to 8 Years. Healthy Eating Research, October 2021.

42. Gago CM, Wynne JO, Moore MJ, Cantu-Aldana A, Vercammen K, Zatz LY, et al. Caregiver Perspectives on Underutilization of WIC: A Qualitative Study. Pediatrics. 2022.

43. Transition to EBT in WIC: Review of Impact and Examination of Participant Redemption Patterns. Altarum Institute.

44. Eriksson N, Stenius M. Changing behavioral patterns in grocery shopping in the initial phase of the COVID-19 crisis—A qualitative study of news articles. 2020.

45. Grashuis J, Skevas T, Segovia MS. Grocery shopping preferences during the COVID-19 pandemic. Sustainability. 2020;12(13):5369.

46. Arm K, Miller L, Tucker AC. Online Grocery Shopping During the COVID-19 Pandemic. January 26, 2022.

47. Trude ACB, Lowery CM, Ali SH, Vedovato GM. An equity-oriented systematic review of online grocery shopping among low-income populations: implications for policy and research. Nutrition Reviews. 2022. doi: 10.1093/nutrit/nuab122.

48. Zhongming Z, Linong L, Wangqiang Z, Wei L. Biden-Harris Administration’s Actions to Reduce Food Insecurity Amid the COVID-19 Crisis. 2021.

49. WIC Program: COVID-19 Emergency Response 2022. Available from: https://www.nutritionnc.com/wic/covid19.htm.

50. Zhang Q, Park K, Zhang J, Tang C. The Online Ordering Behaviors among Participants in the Oklahoma Women, Infants, and Children Program: A Cross-Sectional Analysis. International Journal of Environmental Research and Public Health. 2022;19(3):1805.

51. Biediger-Friedman L, Crixell SH, Silva M, Markides BR, Smith KS. User-centered design of a Texas WIC app: a focus group investigation. American journal of health behavior. 2016;40(4):461-71.

52. Harari N, Rosenthal MS, Bozzi V, Goeschel L, Jayewickreme T, Onyebeke C, et al. Feasibility and acceptability of a text message intervention used as an adjunct tool by WIC breastfeeding peer counsellors: The LATCH pilot. Maternal & child nutrition. 2018;14(1):e12488.

Supporting information captions:

S1 Appendix: 21-item Standards for Reporting Qualitative Research (SRQR) checklist

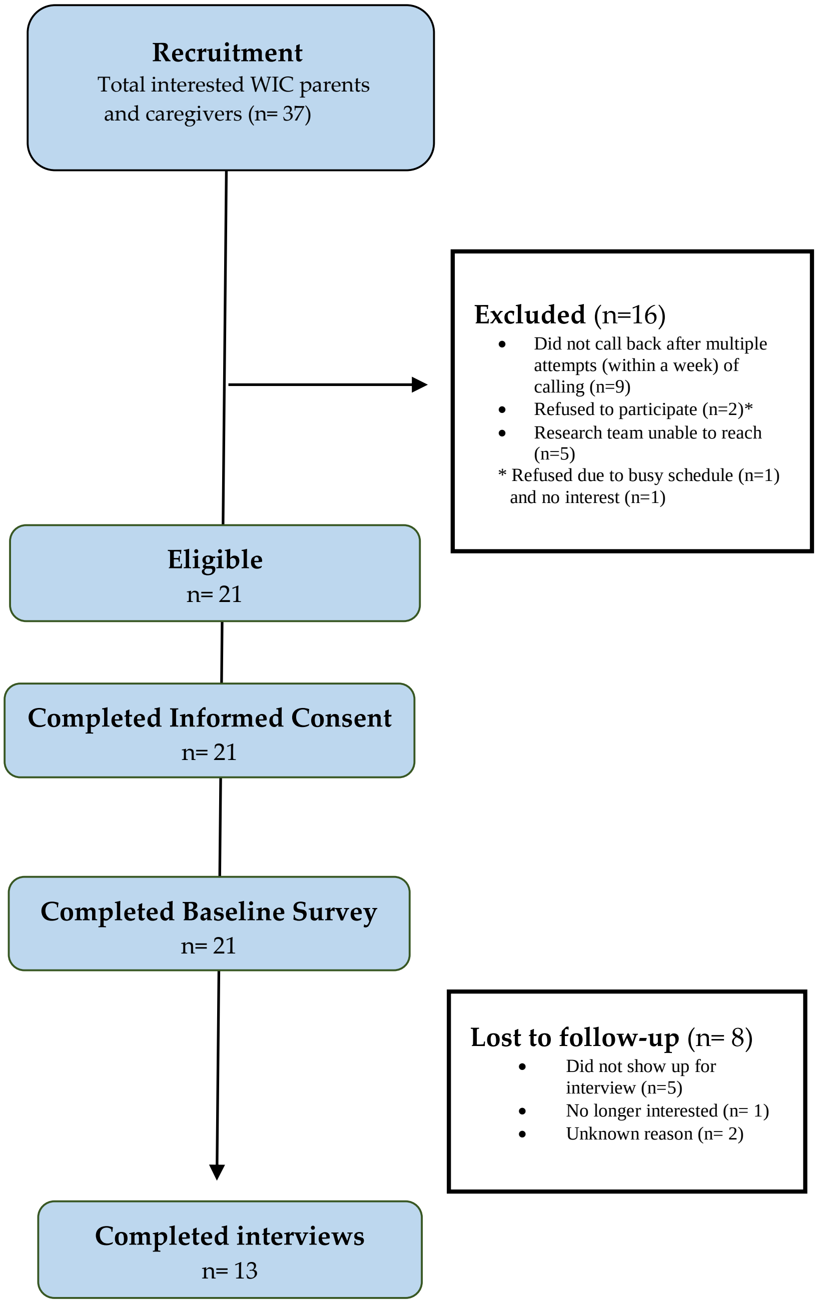

Figure 1: Consort diagram describing flow of participants through the formative research

Click here to access/download

Supporting Information

S1 Appendix SRQR_Checklist.docx
