# Supplementary material for: Perspectives on healthy eating practices and acceptance of WIC-approved foods among parents of young children enrolled in WIC
Source: PLoS One. 2023 Dec 21;18(12):e0295902. doi: 10.1371/journal.pone.0295902 (PMC10734995; doi:10.1371/journal.pone.0295902)
Supplement: Attachment — Submitted filename: plos-coment.docx [file pone.0295902.s003.docx]

Abstract

Make classify your abstract into introduction ,methods, result, and conclusion part.

Nearly 20% of children in the U.S. have obesity, affecting 14.4 million children [1]. 48 Childhood obesity puts children at a higher risk of developing metabolic syndrome, type 49 2 diabetes, kidney diseases, and cardiovascular diseases later in adulthood [2, 3].

Don’t start sentences with problem at least you are expected to mention facts about your study like obesity and dietary practice.

To ensure the intervention is congruent with the needs of WIC parents and 85 caregivers, we conducted in-depth interviews to guide the development. These interviews 86 focused on highlighting barriers and facilitators to consuming WIC-approved foods and 87 behaviors associated with healthy eating. The aim of this paper is to describe findings 88 from the formative research and provide guidance for future intervention development.

Make this part to method part it is not introduction part

Table 2. Characteristics of the participants who completed interviews (n=13). Change to sociodemographic characteristics of the participants who completed interviews

Paper is very interesting
